# Role of urinary pregnancy testing in the diagnosis of men with testicular cancer

**DOI:** 10.1308/rcsann.2023.0029

**Published:** 2024-04-02

**Authors:** L Paramore, AS Chetwood

**Affiliations:** Frimley Health NHS Foundation Trust, UK

## Background

Human chorionic gonadotropin (hCG), alpha fetoprotein and lactate dehydrogenase play important roles in the initial diagnosis, staging and surveillance of seminomatous and non-seminomatous germ cell tumours in testicular cancer.^1^ There can be a significant delay (measured in days) in obtaining a serum hCG result from our local laboratory.

## Technique

Modern urine hCG testing strips provide a rapid result and can be undertaken at the point of care. Siemens CLINITEST Pregnancy hCG testing strips (Siemens Healthineers, Tarrytown, NY, USA) show a positive result when hCG levels in the urine are >25 milli-international units per millilitre (mIU/ml) and a borderline result if hCG levels are 6–25mIU/ml.^2^

## Discussion

Urine hCG can be correlated with imaging findings and aid decision-making while awaiting a formal serum result to be used in the ongoing care of patients. It should be noted that hCG is only elevated in fewer than 40% of germ cell tumours^3^ and a negative result does not exclude a testicular malignancy. However, we have recently used this technique in two patients presenting acutely with testicular pain, but with suspicious testicular examination and ultrasound imaging. A positive urine hCG result helped aid decision-making regarding the timing and approach of surgery. A radical inguinal orchidectomy was undertaken during the same presentation for these two patients following staging imaging with a computed tomography thorax, abdomen and pelvis scan. We recognise the role of urinary hCG testing in environments with limited access to blood testing and its value in timely decision-making in all environments.
